# Molecular detection of tick-borne pathogens in cattle from Southwestern Ethiopia

**DOI:** 10.1371/journal.pone.0188248

**Published:** 2017-11-20

**Authors:** Zerihun Hailemariam, Jürgen Krücken, Maximilian Baumann, Jabbar S. Ahmed, Peter-Henning Clausen, Ard M. Nijhof

**Affiliations:** 1 Freie Universität Berlin, Institute for Parasitology and Tropical Veterinary Medicine, Berlin, Germany; 2 College of Veterinary Medicine, Haramaya University, Dire Dawa, Ethiopia; 3 Freie Universität Berlin, FAO Reference Center for Veterinary Public Health, Berlin, Germany; University of Queensland, AUSTRALIA

## Abstract

Tick-borne diseases (TBDs) cause significant losses among livestock and impact the livelihoods of resource-poor farming communities worldwide. In Ethiopia, detailed studies on the epidemiology of tick-borne pathogens (TBPs) in cattle using sensitive molecular detection methods are scarce. The objective of this study was to determine the prevalence and species composition of bovine TBPs of veterinary significance in local cattle populations. A comprehensive cross-sectional epidemiological study was conducted in cattle populations of Illubabor zone in Southwestern Ethiopia from June to August 2013. For this purpose, blood samples were collected from 392 cattle. A combination of polymerase chain reaction (PCR) and a Reverse Line Blot (RLB) hybridization assay was employed for the detection of TBPs in these samples. The PCR/RLB results of the 392 blood samples indicated a high overall prevalence of 96.9% for TBPs, including *Theileria mutans* (66.1%), *Theileria orientalis* (51.8%), *Anaplasma* sp. Omatjenne (25.5%), *Anaplasma marginale* (14.5%), *Babesia bigemina* (14.0%) and *Theileria velifera* (13.0%) and minor occurrences of *Ehrlichia ruminantium* (0.5%) and *Ehrlichia minasensis* (0.26%). Moreover, three novel *Anaplasma* genotypes were detected in bovine blood samples. A phylogenetic analysis revealed that they most likely represent three, but at least two, new species. The prevalence of the three novel *Anaplasma* species, preliminary designated as *Anaplasma* sp. Hadesa, *Anaplasma* sp. Saso and *Anaplasma* sp. Dedessa, was 12.5%, 14.3% and 5.6%, respectively. Overall, a total of 227 cattle (57.9%) were found to be co-infected with two or more TBPs simultaneously and 86 different species combinations were observed. The findings show a very high burden of infection of cattle with TBPs in Ethiopia. The high frequency of co-infections suggests that clinical manifestations might be complex. Further research is required to determine the pathogenicity, host cell types and vector of the three novel *Anaplasma* species identified in this study.

## Introduction

With approximately 54 million heads of cattle, Ethiopia is considered to have the largest livestock population in Africa. The majority of the population is comprised of local zebu breeds, but the numbers of imported *Bos taurus* breeds and their crosses are increasing [1]. In Ethiopia, cattle are infested by several species of hard ticks (Acari: Ixodidae) including *Amblyomma cohaerens*, *Amblyomma variegatum*, *Rhipicephalus decoloratus*, *Rhipicephalus pulchellus*, *Rhipicephalus evertsi evertsi*, *Rhipicephalus praetextatus* and *Hyalomma rufipes* [2]. These ticks can act as vectors for a variety of pathogens with veterinary and zoonotic importance and several tick-borne diseases (TBDs) are known to be endemic in Ethiopia. This includes bovine babesiosis caused by *Babesia bigemina* and *Babesia bovis*, bovine anaplasmosis caused by *Anaplasma marginale* and heartwater caused by *Ehrlichia ruminantium*. In addition, a number of mildly pathogenic *Theileria* species such as *Theileria mutans*, *T*. *velifera*, and *T*. *orientalis* have also been reported to occur [3–6]. In indigenous breeds of cattle, the course of these TBDs is usually subclinical. However, they pose a greater challenge to susceptible exotic breeds of cattle, thus representing a major constraint in the upgrading and development of cattle production in Ethiopia [7].

Given the importance of cattle husbandry in Ethiopia, information regarding the prevalence and species composition of bovine tick-borne pathogens (TBPs) is of paramount importance. However, studies on the epidemiology of these pathogens in Ethiopia are remarkably scarce. Accordingly, this study aimed to investigate the occurrence of bovine TBPs of veterinary significance in Southwestern Ethiopia. Due to the existence of diverse species of TBPs known to be transmitted by hard ticks in Ethiopia, this study relied on a combination of PCR and a Reverse Line Blot (RLB) hybridization assay for the simultaneous detection of TBPs from bovine blood samples.

## Materials and methods

### Study area

**Fig 1:**
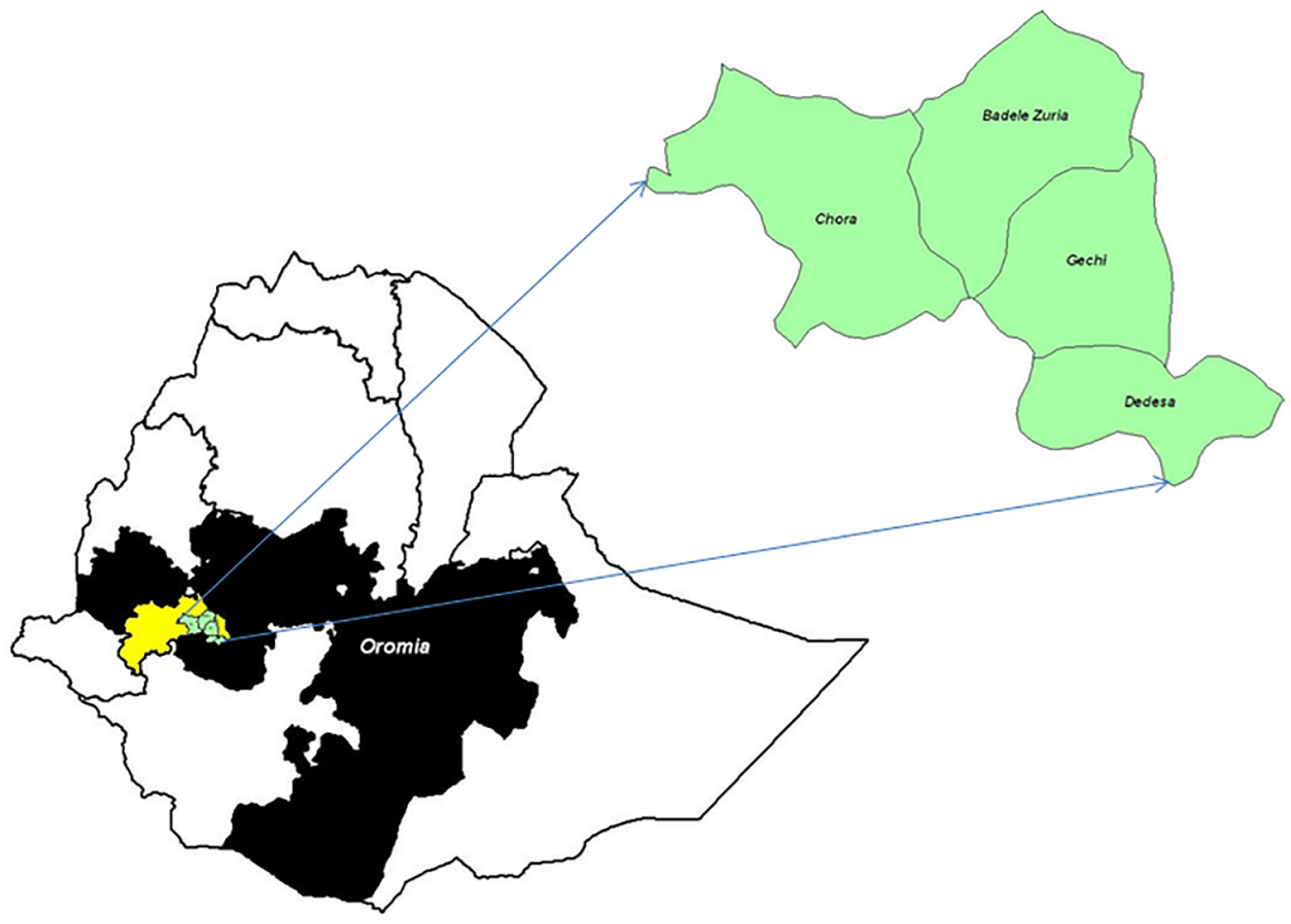
Map of Ethiopia with the study area. Districts studied indicated in green color and Illubabor zone of Oromia region indicated in yellow color.

The study was conducted in Illubabor zone of Oromia Regional State, Southwestern Ethiopia (Fig 1). Illubabor zone is located at 7°27′40″ to 9°2′10″ North and 34°52′12″ to 41°34′55″ East. The zone has 1.6 million hectares of land with altitudes ranging from 500 to 2575 meter above sea level (m. a.s.l), 10% of which is highland (2500–2575 m a.s.l.), 67% is midland (1500–2500 m a.s.l.) and 23% is lowland (500–1500 m a.s.l.). The temperature varies from 18°C to 24°C and annual precipitation ranges from 1500–2200 mm, with 6 to 9 months of rainfall. Illubabor zone is divided into 24 administrative districts of which 2 are urban and 22 are rural. Approximately 1.6 million persons live in the region, of which 88% lives in the rural areas. Agriculture is the mainstay of the economy with mixed farming system practiced at subsistence level. The size of the cattle population in the zone is approximately 1.3 million [8].

### Study design, sample size and sampling strategies

A cross-sectional study design was employed to address the objective of this study. The sample size was determined using the online Epitools epidemiological calculators [9]. The level of confidence and absolute precision desired were set to 95% and 0.05, respectively while the expected (unknown) prevalence was set to 50%. Having stratified the study population by districts (4) and peasant associations (PAs) (12), proportional allocation was utilized to determine the number of cattle to be sampled per stratum. Combinations of stratified, multistage and purposive sampling methods were applied following previously published guidelines [10, 11]. Four districts in the Illubabor Zone were selected purposively based on previous history of occurrence of TBDs (first stage). Then a list of PAs within districts was compiled from data obtained from the districts’ agricultural offices (second stage) and three sampling PAs from each district were randomly selected using a lottery system. Villages in each PA were selected in collaboration with the respective district’s animal health personnel by purposive sampling based on farmers’ cooperation, logistics and share of communal grazing land (third stage). From selected villages, herds grazing within the same grazing land were considered as primary sampling unit. Then cattle were sampled randomly from each grazing herd.

### Sample collection and processing

The sampling was carried out in July and August 2013. All cattle included in the study were selected and sampled with the consent of their owners and chiefs of the villages. Approximately 4 ml of blood was collected from each selected animal by jugular venipuncture in ethylenediaminetetraacetic acid (EDTA) containing Vacutainer tubes (BD Biosciences, Franklin Lakes, NJ, USA). Blood samples were also collected from an ear vein pierced with a lancet after which blood was drained into 75 mm×1.5 mm heparin-treated haematocrit capillary tube for the measurement of packed cell volume (PCV). A drop of blood from the ear vein was also used to prepare thin blood smears. Ethical approval for this study was obtained from the Research and Ethics Committee of the College of Veterinary Medicine, Haramaya University, Ethiopia (Ref.: CVM/76/13). Informed consent was obtained from all livestock owners included in the study.

A standardized questionnaire was used to obtain information regarding farm management practices and possible risk factors associated to infection with TBPs. The questionnaires comprised questions regarding the type of acaricide used, timing and frequency of acaricide application, drug administered to treat clinical cases of TBDs and the farmers’ knowledge about ticks and TBDs.

Initial sample processing was conducted at Bedelle regional veterinary laboratory, Ethiopia. Upon reaching the laboratory, 125 μl of EDTA blood was spotted on Flinders Technology Associates (FTA) Classic^®^ cards (FTA cards, Whatman Biosciences, Cambridge, UK) and allowed to air-dry over night at room temperature.

Air-dried thin blood smears were fixed in methanol for 5 min and stained with 10% Giemsa’s stain (Merck, Darmstadt, Germany) for 45 min. At least 50 microscopic fields were examined per slide at 1000× magnification (oil immersion). Presence of tick-borne haemoparasites was recorded; identification was carried out to the genus and, where possible, the species level [12].

Blood samples in microhematocrit capillary tubes were centrifuged using a microhaematocrit centrifuge (Hawksley, Sussex, UK) at 12,000 rpm for 5 min. The PCV was measured using a microhaematocrit reader (Hawksley, Sussex, UK). Animals with a PCV lower than 24% were considered anemic.

### DNA extraction from FTA cards

DNA was extracted from FTA cards following a previously described protocol [13]. Briefly, sixteen 3 mm diameter discs of each sample were punched out using a Harris Micro-Punch (Whatman) and placed into 1.5 ml Eppendorf tubes. In order to avoid carryover contamination between samples, discs were cut from a blank filter paper after each sample. As negative extraction controls, discs were punched from blank FTA cards and processed together with the other samples. The FTA discs were washed and prepared using FTA purification reagent following Whatman Protocol BD08. After drying at 45°C for at least 60 min, discs were incubated at 90°C in 100 μl of 5% (w/v) aqueous suspension of Chelex^®^ 100 resin for 30 min. This was followed by centrifugation of the sample at 20,000 × g for 3 min. The supernatant was subsequently transferred to a new sterile pre-labelled microcentrifuge tube and used as a template for the PCR.

### Molecular detection of tick-borne pathogens

Amplification of a fragment of 460–540 bp from the 18S SSU ribosomal ribonucleic acid (rRNA) gene spanning the V4 region of *Babesia* and *Theileria* species was carried out with forward primer, RLB-F2 and reverse primer RLB-R2 [14]. A second PCR was performed using forward primer Ehr-F2 (5′-AGA GTT TGA TCC TGG CTC AG-3′) and reverse primer Ehr-R2 (5′-biotin-GAG TTT GCC GGG ACT TYT TCT-3′) amplifying a fragment of 460–500 bp from the V1 hypervariable region of the rickettsial 16S rRNA gene [13]. The PCR reactions were performed in a 25 μl reaction volume consisting of 0.2 mM dNTPs (Thermo Fisher Scientific), 0.5 μM of each primer, 0.02 U/μl Phusion Hot Start II High Fidelity DNA Polymerase (ThermoFisher Scientific) and 2.5 μl template DNA in 1 × Phusion HF buffer (Thermo Fisher Scientific). The *Babesia/Theileria* PCR cycle parameters included an initial denaturation at 98°C for 30 s, followed by 10 cycles of 98°C for 10 s, 68°C for 20 s, 72°C for 15 s, with lowering of the annealing step after every second cycle by 2°C until an annealing temperature of 58°C was reached. The reaction was followed by 40 cycles with annealing at 58°C for 20 s before a final extension at 72°C for 8 min was conducted. The annealing temperature in the *Ehrlichia/Anaplasma* PCR cycle started at 71°C and was gradually lowered to 61°C, all other conditions were similar to the *Babesia/Theileria* PCR protocol. Gel electrophoresis and RLB were performed as previously described [13]. A list of RLB probes used for detecting pathogen DNA in this study is presented in Table 1.

**Table 1:** List of RLB probes used in this study.

Species	Sequence (5’-3’)	Reference
*Anaplasma bovis*	GTAGCTTGCTATGAGAACA	[15]
*Anaplasma centrale*	TCGAACGGACCATACGC	[15]
*Anaplasma marginale*	GACCGTATACGCAGCTTG	[15]
*Anaplasma* sp. Dedessa	ACGGATTATATTTGTAGCTTGCT	this study
*Anaplasma* sp. Hadesa	AGCTTGCTACAGAAGTAATTAGTGG	this study
*Anaplasma* sp. Omatjenne	CGGATTTTTATCATAGCTTGC	[15]
*Anaplasma* sp. Saso	GTCGAACGGATTTTTATCATAGC	this study
*Babesia bigemina*	CGTTTTTTCCCTTTTGTTGG	[16]
*Babesia bovis*	CAGGTTTCGCCTGTATAATTGAG	[16]
*Babesia caballi*	GTGTTTATCGCAGACTTTTGT	[17]
*Babesia* genus specific 2	ACTAGAGTGTTTCAAACAGGC	[18]
*Babesia* genus-specific 1	ATTAGAGTGTTTCAAGCAGAC	[18]
Bacteria catch-all	CTACGGGAGGCAGCAGT	
*Ehrlichia / Anaplasma* genera specific	TTATCGCTATTAGATGAGCC	[19]
*Ehrlichia canis*	TCTGGCTATAGGAAATTGTTA	[19]
*Ehrlichia chaffeensis*	ACCTTTTGGTTATAAATAATTGTTA	[19]
*Ehrlichia minasensis*	CGGACAATTATTTATAGCTTTTGGC	this study
*Ehrlichia ruminantium*	AGTATCTGTTAGTGGCAG	[15]
*Midichloria* genus-specific	GCGAAATAACAGTTGGAAGCAAT	this study
*Rickettsia aeschlimanni*	ATATTATACTGTATGTAGCCCC	[20]
*Rickettsia africae*	ACTAATTTTTGGGGCTTGCTC	this study
*Rickettsia* catch-all	TAGCTCGATTGRTTTACTTTG	[20]
*Rickettsia conorii*	GTTATATACTGTAGCCCTG	[20]
*Rickettsia massiliae*	CCGCCACGATATCTAGAAAAATTA	this study
*Theileria / Babesia* genera specific	CTGTCAGAGGTGAAATTCT	[16]
*Theileria annulata*	CCTCTGGGGTCTGTGCA	[21]
*Theileria equi* A1	TTGGCGTTTGTCATCGTTGC	this study
*Theileria equi* A2	GTTGTGGCTTAGTTGGGGCAT	this study
*Theileria equi* B	CTGTATCGTTATCTTCTGCTTGACA	this study
*Theileria* genus-specific	ATTAGAGTGCTCAAAGCAGGC	[18]
*Theileria lestoquardi*	ATTGCTTGTGTCCCTCCG	[22]
*Theileria mutans*	CTTGCGTCTCCGAATGTT	[16]
*Theileria orientalis*	GGCTTATTTCGG(AT)TTGATTTT	[16]
*Theileria ovis*	TTGCTTTTGCTCCTTTACGAG	[22]
*Theileria parva*	TCGGACGGAGTTCGCTTTG	this study
*Theileria separata*	GGTCGTGGTTTTCCTCGT	[22]
*Theileria* sp. (buffalo)	CAGACGGAGTTTACTTTGT	[23]
*Theileria* sp. (sable)	GCTGCATTGCCTTTTCTCC	[24]
*Theileria taurotragi*	TCTTGGCACGTGGCTTTT	[16]
*Theileria velifera*	CCTATTCTCCTTTACGAGT	[16]

### DNA purification and confirmation of RLB positive samples by sequencing

The 16S or 18S rRNA genes of selected RLB-positive samples were amplified and sequenced to verify the RLB results. For *Babesia* species, a ~550 bp fragment of the 18S rRNA gene was amplified using primers Babesia specific-F (5’-CCA TCA GCT TGA CGG TAG GG-3’) and RLB-R2 with the Babesia/Theileria RLB PCR protocol described above. The same protocol and cycle parameters were also used for the amplification of a ~560 bp fragment of the *Theileria* 18S rRNA gene with primers Theileria specific-F (5’-CTA TCA GCT TTG GAC GGT AGG G-3’) and RLB-R2. For confirmation of RLB positive *Anaplasma* and *Ehrlichia* samples, a ~1438 bp fragment of 16S rRNA gene was amplified using forward primer Ehr-F2 and reverse primer AnaEhrl full (5′-CCC TAG TCA CTR ACC CAA CCT TA-3′). PCR cycle parameters included an initial denaturation at 98°C for 60 s, followed by 40 cycles of 98°C for 10 s, 61.5°C for 10 s and 72°C for 45 s before a final extension at 72°C for 10 min. The *Anaplasma* and *Ehrlichia* samples were sequenced with reverse primer Ehr-R4 (5′-GAG TTW GCC GGG RCT TYT TCT-3′).

DNA was cleaned directly from the PCR reactions with the DNA Clean & Concentrator^TM^-5 Kit (Zymo Research Corporation, Irvine, USA) or from excised gel bands by the Zymoclean^TM^ Gel DNA Recovery Kit (Zymo Research Corporation) according to the manufacturer’s instructions. Purified products were sequenced by LGC Genomics GmbH (Berlin, Germany).

### DNA cloning, sequencing and phylogenetic analysis

For characterization of novel *Anaplasma* species, amplification products from positive samples were analyzed by agarose gel electrophoresis and expected bands were excised and purified by the Gel DNA recovery kit (Zymo Research). Purified PCR products were cloned into the StrataClone blunt-end PCR cloning vector ‘pSC-B-amp/kan’ supplied in the StrataClone Blunt PCR cloning kit (Agilent Technologies, CA, USA) and recombinant plasmid vectors were transformed into Solopack^®^ competent cells (Agilent Technologies, CA, USA) according to the manufacturer's instructions. Following plasmid DNA isolation using the Plasmid Mini Prep Kit EasyPrep® Pro (Biozym, Oldendorf, Germany), clones with inserts were sequenced by LGC Genomics (Berlin). The 16S rRNA clones were sequenced bidirectionally. The obtained sequences were analyzed by BLASTn search (https://blast.ncbi.nlm.nih.gov/Blast.cgi).

Selected small ribosomal (16S) rRNA sequences from representative members of the family Anaplasmataceae were aligned using MAFFT 7 with the Q-INS-i iterative refinement method which considers RNA structure predictions [25]. Phylogenetic maximum-likelihood analysis was conducted with RAxML 8.2.9 [26] on the CIPRES gateway [27]. The GTRGAMMA model was chosen with 25 substitution rate categories and a rapid bootstrap analysis (1000 replicates) with identification of the tree with the highest likelihood in the same run was performed. The resulting tree was used as additional input in a second run to constrain the tree topology and calculate alternative node support values using the Shimodaira–Hasegawa modification of the likelihood ratio test. The tree was visualized using Mega 7 [28] and rooted using the sequences from species outside of the genus *Anaplasma*. Novel *Anaplasma* 16S rRNA sequences obtained in this study were deposited in the GenBank database with accession numbers KY924884—KY924886.

### Statistical analysis

Software used for statistical analysis in this study were IBM SPSS Statistics Version 23.0 and OpenEpi Version 3.03. The prevalence of several pathogens and 95% confidence intervals were calculated as Wilson Score intervals. Univariate analysis of associations using the Chi-squared test was carried out for each exposure variable, with the RLB based infection prevalence by *B*. *bigemina* and *A*. *marginale*, infections considered as a binary outcome (positive or negative). We tried to perform multivariate analysis for risk factor analysis but that was not applicable due to the co-linearity of many variables. The main consideration taken into account to perform risk factor analysis only for *B*. *bigemina* and *A*. *marginale* were their economic significance and availability of sufficient data to perform a risk factor analysis. P values less than 0.05 were considered significant. The exposure variables considered were age, sex, breed, management system, acaricide used and frequency of acaricide applications.

## Results

### Demography of the study population

Blood samples were collected from a total of 392 apparently healthy cattle in Illubabor zone, Southwestern Ethiopia. Samples were collected from 12 PAs in four districts. The majority of the animals were local Zebu breeds (*Bos taurus*) (371/392; 94.6%), and the rest (21/392; 5.4%) Holstein Friesian x Zebu crossbreds. While the local zebu breeds of cattle were kept under extensive managements system, all cross-bred cattle was managed semi-intensively, i.e. they received supplementary feed in addition to grazing. The study population comprised more females (60.5%, 237/392) than males 39.5% (155/392) (Table 2).

**Table 2:** Descriptive statistics and univariable analysis of risk factors associated with *B*. *bigemina* and *A*. *marginale* infections detected by RLB in cattle from Southwestern Ethiopia.

Variable	Categories	Total No. (%)	*B*. *bigemina*		*A*. *marginale*	
			No. + ve (%)	p-value	No. + ve (%)	p-value
Breeds	Zebu	371 (94.6)	50 (13.5)	0.194	47 (12.7)	<0.001*
	Cross-bred	21 (5.4)	5 (23.8)		10 (47.6)	
Sex	Male	155 (39.5)	25 (16.1)	0.37	19 (12.3)	0.38
	Female	237 (60.5)	30 (12.7)		38 (16.0)	
Age	Calf	51 (13.0)	9 (17.6)	0.70	5 (9.8)	0.34
	Young	120 (30.6)	17 (14.2)		15 (12.5)	
	Adult	221 (56.4)	29 (13.1)		37 (16.7)	
Management system	Semi-intensive	21 (5.4)	5 (23.8)	0.19	10 (47.6)	<0.001*
	Extensive	371 (94.6)	50 (13.5)		47 (12.7)	
Acaricide used	Diazinone	170 (43.4)	26 (15.3)	0.28	32 (18.8)	0.06
	Cyper/deltamethrin	52 (13.3)	4 (7.7)		7 (13.5)	
	Amitraz	144 (36.7)	19 (20.2)		18 (12.5)	
	Ivermectin	26 (6.6)	6 (23.1)		0 (0)	
Frequency of acaricide application	1–3× per year	329 (83.9)	48 (14.6)	0.09	40 (12.2)	<0.001*
	4–6× per year	42 (10.7)	2 (4.8)		7 (16.7)	
	6–8× per year	21 (5.4)	5 (23.8)		10 (47.6)	

*As all the 21 cattle managed semi-intensively and sprayed four to six times per year with acaricide are the same 21 cross-bred animals in the breed category, the statistically significant association observed here was not taken as a valid association.

No.: Number, + ve: Positive.

### Microscopic identification of tick-borne haemoparasites

Microscopic examination of Giemsa-stained blood smears revealed that 67 out of 392 (17.1%) samples were positive for at least one TBP. The most frequently observed haemoparasites were *Theileria* spp. (39/392; 10.0%), followed by *A*. *marginale* (18/392; 4.6%), *B*. *bigemina* (5/392; 1.3%), mixed infection of *Theileria* spp. and *A*. *marginale* (3/392; 0.8%) and mixed infection of *B*. *bigemina* and *A*. *marginale* (2/392; 0.5%).

### RLB based prevalence of hemoparasites

Out of 392 blood samples applied on FTA cards examined for TBPs by PCR/RLB, 380 samples (96.9%) were positive for at least one hemoparasite. DNA from eleven different TBPs including three novel *Anaplasma* species was detected. Among the TBPs detected were *T*. *mutans* (259/392; 66.1%), *T*. *orientalis* (203/392; 51.8%), *Anaplasma* sp. Omatjenne (100/392; 25.5%), *A*. *marginale* (57/392; 14.5%), *B*. *bigemina* (55/392; 14.0%) and *T*. *velifera* (51/392; 13.0%), with minor occurrences of six other haemoparasites including the highly pathogenic *E*. *ruminantium*. Another recently identified pathogenic *Ehrlichia* species, *Ehrlichia minasensis* was detected for the first time in Ethiopia (1/392; 0.3%). The *E*. *minasensis* sequence generated in this study was 100% (420/420) identical with the *E*. *minasensis* genotype UFMG–EV 16S rRNA gene sequence deposited in GenBank (JX629805).

When a number of the PCR products were sequenced to confirm the RLB results, three samples showed a mixed sequence content, indicating the presence of multiple sequences. These PCR products were subsequently cloned and re-sequenced. A preliminary BLASTn analysis of these sequences revealed the presence of three novel 16S rRNA sequences that did not show 100% identity to any known GenBank entry. They were preliminary designated as *Anaplasma* sp. Hadesa, *Anaplasma* sp. Saso and *Anaplasma* sp. Dedessa. Novel RLB probes were developed based on these sequences and all Anaplasma/Ehrlichia positive samples were screened again by RLB on a membrane that included these new probes. The prevalence of the three novel *Anaplasma* genotypes was thus determined to be 49/392 (12.5%), 56/392 (14.3%) and 22/392 (5.6%), respectively (Table 3).

**Table 3:** Prevalence of tick-borne pathogens in cattle blood samples from Southwest Ethiopia as determined by RLB.

Species	Total (n = 392)	Prevalence (%)	95% CI
*A*. *marginale*	57	14.5	11.40–18.37
*Anaplasma* sp. Omatjenne	100	25.5	21.45–30.05
*E*. *ruminantium*	2	0.5	0.14–1.84
*E*. *minasensis*	1	0.3	0.05–1.43
*B*. *bigemina*	55	14.0	10.94–17.82
*T*. *mutans*	259	66.1	61.25–70.58
*T*. *orientalis*	203	51.8	46.85–56.69
*T*. *velifera*	51	13.0	10.04–16.70
*Anaplasma* sp. Hadesa	49	12.5	9.60–16.14
*Anaplasma* sp. Saso	56	14.3	11.17–18.10
*Anaplasma* sp. Dedessa	22	5.6	3.74–8.35

CI: confidence interval.

### Phylogenetic analysis of novel *Anaplasma* spp

**Fig 2:**
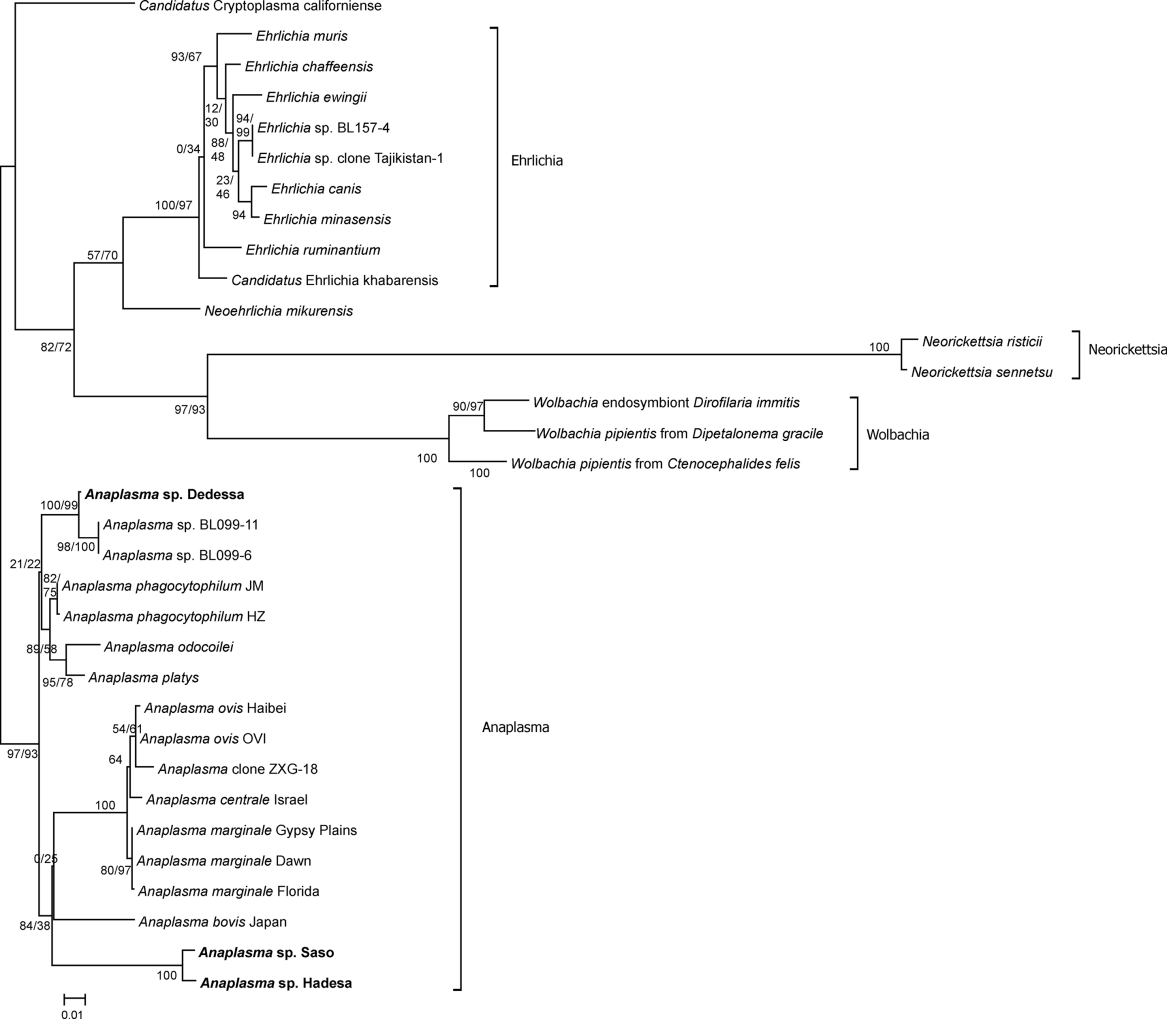
Phylogenetic analysis of *Anaplasma* species identified in this study. Maximum-likelihood phylogenetic analysis of Anaplasmataceae using 16S rRNA sequences. The sequences from other genera in the family Anaplasmataceae were included to serve as outgroup. Numbers before and after the slashes represent node support values obtained by the Shimodaira–Hasegawa likelihood ratio test and bootstrapping, respectively. The scalebar represents 0.01 substitutions per side. The sequences obtained in the present study are highlighted in bold.

Samples containing *Anaplasma* sp. Hadesa, *Anaplasma* sp. Saso and *Anaplasma* sp. Dedessa were used as templates for the PCR-amplification of a ~1438 bp 16S rRNA gene fragment, followed by cloning, sequencing and a phylogenetic analysis. BLASTn searches showed highest 16S rRNA sequence similarity for *Anaplasma* sp. Hadesa to *Anaplasma phagocytophilum* strain HN (KC470064, 1369/1441 bp, 95.0%). *Anaplasma* sp. Saso showed most 16S rRNA sequence similarity with uncultured *Anaplasma* species isolated from canine blood in the Philippines (KP006398, 1372/1440 bp, 95.3%). *Anaplasma* sp. Dedessa showed highest sequence similarity to *Anaplasma* sp. BL099-6 (KJ410247, 1406/1417 bp, 99.2%) originally isolated from *Hyalomma* ticks in China. Comparisons between the three novel *Anaplasma* genotypes revealed 99.1% identity between *Anaplasma* sp. Hadesa and *Anaplasma* sp. Saso. In contrast, identity of *Anaplasma* sp. Dedessa to the other two genotypes was only 95%. In comparison, identity between the three closely related species *A*. *marginale*, *A*. *centrale* and *A*. *ovis* was between 99.3 and 99.5%. For the phylogenetic analysis, only the 16S rRNA gene was included since all three novel *Anaplasma* genotypes occurred as mixed infections and it was not possible to unequivocally pair the 16S rRNA genes with additional genes belonging to the same *Anaplasma* species. The analysis identified all three new genotypes with high support values as members of the cluster formed by the genus *Anaplasma*. *Anaplasma* sp. Dedessa could be identified with high confidence as a closely related relative of *Anaplasma* sp. BL099-6. In contrast, the position of *Anaplasma* sp. Hadesa and *Anaplasma* sp. Saso, which are more closely related to each other than to any other member of the genus *Anaplasma* included in the analysis, could not be ascertained with confidence (Fig 2). Branch lengths between these genotypes (0.012 substitutions/site) are nearly twice as long as between *A*. *marginale* and *A*. *ovis* (0.00637) and 33–45% longer than for the comparisons of *A*. *centrale* with *A*. *ovis* (0.00818 substitutions/site) and *A*. *centrale* with *A*. *marginale* (0.00899 substitutions/site) suggesting that they might represent two closely related species and not only genotypes of the same species.

### Co-infections analysis

A total of 227 cattle (57.9%) were found to be simultaneously co-infected with two or more TBPs. Overall, 86 different species combinations were observed. The level of co-infections ranged from double to sextuple. The majority of the mixed infections occurred as double infections (97/227; 42.7%). The most frequent co-occurrences included *T*. *orientalis* and *T*. *mutans*, *B*. *bigemina* and *T*. *mutans*, *T*. *orientalis* and *T*. *velifera*, and *Anaplasma sp*. *Omatjenne* and *T*. *orientalis* (Table 4 and S1 Table).

**Table 4:** Observed level and frequency of co-infections by different species of tick-borne pathogens.

Level of co-infections	Frequency	%	No of species combinations
Double	97	42.7	12
Triple	56	24.7	26
Quadruple	38	16.7	22
Quintuple	30	13.2	20
Sextuple	6	2.6	6
**Overall**	**227**	**100**	**86**

### Risk factor analysis

Univariate analysis of potential risk factors revealed that breed of cattle was significantly associated with *A*. *marginale* infection (p < 0.05) (Table 2). Cross-bred cattle (47.6%, Odds ratio [OR] = 6.23, 95% CI [2.52; 15.56]) were more likely to be infected with *A*. *marginale* than local Zebu breed cattle. The same 21 animals were also the only one kept under a semi-intensive management system (OR = 6.23) and that were regularly treated with acaricides. Due to this fact, these variables were in the same or a similar way associated with an increased odd to be positive for *A*. *marginale*. Although the difference in prevalence of *B*. *bigemina* infection did not significantly differ compared to local zebu breeds (p > 0.05), a higher proportion of infection with *B*. *bigemina* was also observed in cross-bred cattle.

## Discussion

In this study, a combination of PCR and a RLB hybridization assay was employed for the simultaneous detection of TBPs from bovine blood samples collected on FTA cards. The use of FTA cards facilitated the collection, storage and shipment of the blood samples and was also in compliance with German customs import regulations, which prohibits the import of whole blood samples from Foot and Mouth Disease endemic countries such as Ethiopia. A thorough evaluation of six DNA extraction methods from blood spotted on FTA cards was previously conducted to ensure the use of an optimal extraction method for the analysis of the field samples [13]. However, direct DNA extraction from 200 μl of whole blood using a commercial spin column based method was shown to slightly increase the detection limit of the RLB compared to the use of DNA extracted from sixteen 3 mm diameter FTA discs [13]. It is therefore possible that the results reported here still underestimate the true prevalence of TBPs in Ethiopia.

A complex pattern of co-infections was observed in this study. Mixed infections were detected in 226 samples (57.65%) and overall 86 different species combinations were observed. The highest frequency of co-infection was recorded for *T*. *mutans* and *T*. *orientalis* (S1 Table), which were also the most frequently encountered TBPs, with RLB-based prevalence of 66.1% and 51.8%, respectively. In endemic areas, infections with these mildly pathogenic *Theileria* species are usually acquired by calves early in their lifes, after which they remain life-long carriers [29]. This may explain the high prevalence found for both species. At a more generic level, the high frequency of occurrence of mixed infections may increase or decrease the pathogenicity of existing infections. Results of a recent study suggested heterologous protection by mildly pathogenic *Theileria* species against East Coast Fever caused by *T*. *parva*, reducing the severity of infection [30]. However, interactions between different TBP can be much more complex and involve ecological, epidemiological and also clinical aspects [31–33]. For instance, one pathogen might enhance transmission of the other in the ecosystem as observed for *Borrelia burgdorferi* and *Babesia microti* or mutually enhance disease processes and promote disease severity as reported for *B*. *burgdorferi* and both, *B*. *microti* and *Anaplasma phagocytophilum*. Clinical symptoms in co-infected hosts can considerably deviate from typical patterns observed in mono-infected animals, which hampers diagnosis and can lead to treatment failures since only one of the two diseases was recognized.

Besides protozoa, DNA from several bacteria was detected by RLB, including *A*. *marginale* (14.5%) and *Anaplasma sp*. *Omatjenne* (25.5%). *Anaplasma marginale* is known to be pathogenic to domestic ruminants, especially in high producing dairy cattle. Under mixed farming systems, serological investigations elsewhere in East Africa showed seroprevalences of 58% for Mbeere District, Kenya [34], 57% for Soroti District, Uganda [35] and 50% in Central Equatoria State, South Sudan [36]. The prevalence of *A*. *marginale* reported in this study is lower compared to the above mentioned results. This is presumably due to the fact that RLB detects active infections or carrier animals, whereas serology cannot differentiate between active and past infection. Cross-reactions between pathogens of the *Anaplasma* genus on serological assays have also been reported and may lead to an over-estimation of the true prevalence when performing a serological screening only [37]. This study also confirms the occurrence of *Anaplasma* (formerly *Ehrlichia*) sp. Omatjenne in Ethiopian cattle. This bacteria was recently also identified in *Am*. *variegatum* and *Am*. *lepidum* ticks collected from two locations in Central Oromia and one in the Amhara Region of Ethiopia [5]. Its pathogenicity is still poorly understood.

Only 2 blood samples from cattle (0.5%) tested positive for *E*. *ruminantium*, which is the first molecular detection of *E*. *ruminantium* in bovine blood samples in Ethiopia. The low apparent prevalence detected here might be attributed to the biology of *E*. *ruminantium*, as it mainly resides in endothelial cells and is only periodically found in the bloodstream [38, 39]. DNA of *E*. *ruminantium* was previously also detected in *Amblyomma* ticks from Ethiopia [5, 6]. The presence of pathogenic TBPs such as *E*. *ruminantium*, *A*. *marginale* and *B*. *bigemina* together with vector tick species [40] should be taken into account when attempting livestock improvement through the introduction of exotic cattle breeds, such as highly productive, taurine (i.e. *Bos taurus*) and other naive breeds in the area.

A new *Ehrlichia* genotype, *Ehrlichia* sp. UFMG-EV was identified in *Rhipicephalus microplus* ticks in Brazil [41]. In 2014, another genotype, *Ehrlichia* sp. UFMT-BV was also detected in cattle, and was subsequently shown to cause clinical symptoms similar to those of canine ehrlichiosis in an experimentally infected calf [42]. The genetic characterization of 16S ribosomal RNA (rRNA) and thio-disulfide oxidoreductase (*dsb*) genes showed that both *Ehrlichia* sp. UFMG-EV and *Ehrlichia* sp. UFMT-BV genotypes represent a single species phylogenetically close to *E*. *canis*. Recently, the name *Ehrlichia minasensis* was proposed for this recently identified *Ehrlichia* species [43]. In this study, we have detected *E*. *minasensis* DNA in a bovine blood sample for the first time outside of the Americas. The *E*. *minasensis* sequence generated in this study exhibited 100% (420/420) identity with the *E*. *minasensis* genotype UFMG–EV 16S rRNA gene sequence deposited in GenBank (JX629805). Since this species was previously identified only from *R*. *microplus*, a tick species that to the best of our knowledge has not been reported to occur in Ethiopia, it suggests transmission of *E*. *minasensis* by ticks other than *R*. *microplus*. The significance of this pathogen for bovine health and identification of the vector responsible for its transmission in Ethiopia requires further investigation.

Remarkably, in the present study, three novel *Anaplasma* genotypes were identified from naturally infected cattle from Ethiopia that most likely represent three, but at least two new species. The prevalence of these *Anaplasma* genotypes ranged from 5.6% - 14.3%. Sequence analysis indicated that these organisms are phylogenetically distinct from known *Anaplasma* species. Two of the species, *Anaplasma* sp. Hadesa and *Anaplasma* sp. Saso, are closely related and appear to be distant to any of the other members of the genus *Anaplasma*. The branches separating *Anaplasma* sp. Hadesa and *Anaplasma* sp. Saso are short, but longer than those separating e.g. *A*. *marginale*, *A*. *centrale* and *A*. *ovis*, three bacterial species that also share 99.3–99.5% 16S rRNA sequence identity. *Anaplasma centrale* was initially described as a subspecies of *A*. *marginale* [44] but even comparison of whole genome sequences could not resolve whether *A*. *centrale* should be considered a subspecies of *A*. *marginale* or a separate species [45]. In contrast, validity of the species status for *A*. *ovis* is usually not questioned. The biology of the three species differs in terms of morphology/position of the inclusion bodies in the erythrocyte, vertebrate host spectrum, pathogenicity and tick vector [46] suggesting that they represent independently evolving species. It is therefore likely that *Anaplasma* sp. Hadesa and *Anaplasma* sp. Saso also represent independent, closely related species and not simply two genotypes within one species. It was unfortunately not possible to resolve the phylogenetic position of these bacteria in the genus *Anaplasma* solely on the basis of the 16S rRNA sequence. Additional sequences from other genes and multi-locus phylogenetic analysis using material from mono-infected animals will be required to unravel the questions if these are truly separate species and what the exact phylogenetic position of these species within the genus might be.

Based on the 16S rRNA sequence, *Anaplasma* sp. Dedessa appears to be a previously unrecognized species and a close relative of Anaplasma BL099-6, which was recently characterized as *Candidatus* Anaplasma boleense [47]. Analysis of samples from mono-infected cattle was again not possible due to high prevalence of mixed infection by various *Anaplasma* species.

Certain members of the genus *Anaplasma* are recognized to be important human and animal pathogens [48]. Recently, in addition to *Anaplasma phagocytophilum* and *Anaplasma ovis* that have been recognized as pathogens of human anaplasmosis [49, 50], a novel *Anaplasma* species designated “*Anaplasma capra*” has been identified in goats, ticks and humans in northern China [51]. Moreover, it is assumed that additional *Anaplasma* species remain undiscovered and contribute to human and/or animal diseases [52]. It is unknown whether the *Anaplasma* species detected in this study are pathogenic to humans or livestock animals. Isolation and further characterization of these *Anaplasma* species from infected animals as well as their zoonotic potential need to be further investigated. Moreover, it would be interesting to screen questing ticks in the study area to ascertain tick vectors transmitting these bacteria.

A significant association was observed between cross-breed (in comparison to zebu) cattle and a high prevalence of *A*. *marginale* infection. However, the same or a similar positive effect on *A*. *marginale* prevalence were observed for a semi-intensive management system and highly frequent (six to eight times per year) use of acaricides since these three categories of the variables were completely collinear, i.e. they included the same 21 animals from a single farm. This co-linearity also prevented the use of multi-variate analyses methods and exclusion of the 21 animals identified no significant effects of the remaining variables. Although statistically not significant, compared to local zebu breeds, a higher proportion of infection by *B*. *bigemina* was also observed in the same 21 cross-bred cattle. In this regards, the limitation of this study is that a lower percentage of crossbred cattle were studied compared to local zebu breed. However, this represents the locally available cattle breeds since the system to identify the animals that were included did not consider the breed and was largely random. Future case control studies with equal or at least representative numbers of crossbred cattle are necessary to address higher susceptibility of cross-bred animals to *A*. *marginale* and other TBP. Such studies will also allow to statistically evaluating the effects management practices and acaricide treatment in relation to the breed in the epidemiological situation in Ethiopia and probably many similar regions in the tropics.

In conclusion, this study revealed a very high prevalence of tick-borne pathogens close to 100% in the study area and co-infections were more common than single infections. This might have implications for potential interactions of pathogens and the patterns of clinical symptoms. The significance on animal health, zoonotic potential and vectors responsible for transmission of the novel *Anaplasma* species identified in this study as well as of *E*. *minasensis* need to be further investigated. The epidemiological data from this study will provide significant information on tick-borne diseases in the area and will serve as scientific basis for planning future control strategies.

## Supporting information

S1 Table Combinations of mixed infections observed in FTA cards examined for TBPs by PCR/RLB. (DOCX)
