# Supplementary material for: Molecular detection of tick-borne pathogens in cattle from Southwestern Ethiopia
Source: PLoS One. 2017 Nov 20;12(11):e0188248. doi: 10.1371/journal.pone.0188248 (PMC5695778; doi:10.1371/journal.pone.0188248)
Supplement: S1 Table — (DOCX) [file pone.0188248.s001.docx]

**S2 Table. Combinations of mixed infections observed in FTA cards examined for TBPs by PCR/RLB.**

|  | **Nature of infections** | | | | | | | | | | | |
| --- | --- | --- | --- | --- | --- | --- | --- | --- | --- | --- | --- | --- |
|  | **Single** | | **Double** | | **Triple** |  | **Quadruple** | | **Quintuple** | | **Sextuple** | |
|  | **Sp.** | **Total** | **Spp.** | **Total** | **Spp.** | **Total** | **Spp.** | **Total** | **Spp.** | **Total** | **Spp.** | **Total** |
|  | To | 80 | ToTm | 43 | EoToAh | 6 | EoToTmAs | 5 | AmEoToTmAs | 6 | AmBbToTmAdAs | 1 |
|  | Tm | 65 | BbTm | 12 | EoBbTm | 5 | AmEoBbTm | 4 | EoToTmTvAs | 3 | AmEoBbToTmTv | 1 |
|  | Bb | 6 | TmTv | 9 | EoToTm | 5 | ToTmAhAs | 3 | AmBbTmTvAs | 2 | AmEoTmTvAhAs | 1 |
|  | Am | 1 | EoTo | 8 | AmTmAh | 3 | AmEoTmAh | 2 | EoBbToTmAs | 2 | EoBbTmTvAdAh | 1 |
|  | Eo | 1 | EoTm | 6 | AmToTm | 3 | AmEoToTm | 2 | EoToTmAhAs | 2 | EoBbTmTvAhAs | 1 |
|  |  |  | AmTm | 5 | BbTmTv | 3 | AmToTmAd | 2 | AmBbToTmAs | 1 | EoToTmAdAsAh | 1 |
|  |  |  | TmAh | 4 | EoTmAs | 3 | EoTmTvAh | 2 | AmEoBbTmAs | 1 |  |  |
|  |  |  | AmTo | 3 | EoToAs | 3 | EoToTmAh | 2 | AmEoTmTvAd | 1 |  |  |
|  |  |  | BbTo | 3 | AmEoTm | 2 | EoToTmTv | 2 | AmEoTmTvAs | 1 |  |  |
|  |  |  | EoBb | 2 | AmTmAs | 2 | TmTvAdAh | 2 | AmTmTvAhAs | 1 |  |  |
|  |  |  | AhAd | 1 | AmTmTv | 2 | AmBbTmAs | 1 | AmToTmAdAs | 1 |  |  |
|  |  |  | ToAh | 1 | AmToAs | 2 | AmEoTmAs | 1 | AmToTmTvAs | 1 |  |  |
|  |  |  |  |  | EoTmTv | 2 | AmErEoTm | 1 | EoBbTmAhAs | 1 |  |  |
|  |  |  |  |  | TmTvAs | 2 | AmTmTvAh | 1 | EoBbTmTvAd | 1 |  |  |
|  |  |  |  |  | ToAhAs | 2 | AmToTmTv | 1 | EoBbTmTvAh | 1 |  |  |
|  |  |  |  |  | AmBbTm | 1 | BbTmTvAd | 1 | EoBbTmTvAs | 1 |  |  |
|  |  |  |  |  | AmEoTo | 1 | EoBbTmAh | 1 | EoTmTvAdAh | 1 |  |  |
|  |  |  |  |  | EoBbAs | 1 | EoBbTmAs | 1 | EoTmTvAhAs | 1 |  |  |
|  |  |  |  |  | EoTmAd | 1 | EoBbToTm | 1 | EoToTmTvAd | 1 |  |  |
|  |  |  |  |  | EoTmAh | 1 | EoTmTvAs | 1 | EoToTmTvAh | 1 |  |  |
|  |  |  |  |  | EoToAd | 1 | TmTvAhAs | 1 |  |  |  |  |
|  |  |  |  |  | ErToTm | 1 | ToTmAhAd | 1 |  |  |  |  |
|  |  |  |  |  | TmAhAs | 1 |  |  |  |  |  |  |
|  |  |  |  |  | TmTvAh | 1 |  |  |  |  |  |  |
|  |  |  |  |  | ToTmAh | 1 |  |  |  |  |  |  |
|  |  |  |  |  | ToTmTv | 1 |  |  |  |  |  |  |
| **Total** | **153** | | **97** | | **56** | | **38** | | **30** | | **6** | |

Abbreviations: Am, *A. marginale;* Eo, *Anaplasma* sp. Omatjenne; Bb, *B. bigemina;* Tm, *T. mutans;* To, *T. orientalis;*

Tv, *T. velifera;* Ah, *Anaplasma* sp. Hadesa; As, *Anaplasma* sp. Saso; Ad, *Anaplasma* sp. Dedessa.

**Summary:** Total combinations of mixed infections: 86

Proportions of samples based on nature of infections: negative samples: 12 (3%); single infection: 153/392 (39.0%); mixed infections: 227/392 (57.9%)
